# Scent dog identification of samples from COVID-19 patients – a pilot study

**DOI:** 10.1186/s12879-020-05281-3

**Published:** 2020-07-23

**Authors:** Paula Jendrny, Claudia Schulz, Friederike Twele, Sebastian Meller, Maren von Köckritz-Blickwede, Albertus Dominicus Marcellinus Erasmus Osterhaus, Janek Ebbers, Veronika Pilchová, Isabell Pink, Tobias Welte, Michael Peter Manns, Anahita Fathi, Christiane Ernst, Marylyn Martina Addo, Esther Schalke, Holger Andreas Volk

**Affiliations:** 1 Department of Small Animal Medicine and Surgery, University of Veterinary Medicine Hannover, Hannover, Germany; 2 Research Center for Emerging Infections and Zoonoses, University of Veterinary Medicine Hannover, Hannover, Germany; 3 Department of Physiological Chemistry, University of Veterinary Medicine Hannover, Hannover, Germany; 4 Hörstel, Germany; 5 Department of Respiratory Medicine, Hannover Medical School, Hannover, Germany; 6 Hannover Medical School, Hannover, Germany; 7 Department of Medicine, Division of Infectious Diseases, University Medical-Center Hamburg-Eppendorf, Hamburg, Germany; 8 Department for Clinical Immunology of Infectious Diseases, Bernhard Nocht Institute for Tropical Medicine, Hamburg, Germany; 9 German Center for Infection Research, Hamburg-Lübeck, Borstel-Riems Germany; 10 Central Institute of Medical Service, German Armed Forces, Koblenz, Germany; 11 Bundeswehr School of Dog handling, German Armed Forces, Ulmen, Germany

**Keywords:** COVID-19, SARS-CoV-2, Volatile organic compounds, Scent detection dogs, Olfactory detection, Saliva

## Abstract

**Background:**

As the COVID-19 pandemic continues to spread, early, ideally real-time, identification of SARS-CoV-2 infected individuals is pivotal in interrupting infection chains. Volatile organic compounds produced during respiratory infections can cause specific scent imprints, which can be detected by trained dogs with a high rate of precision.

**Methods:**

Eight detection dogs were trained for 1 week to detect saliva or tracheobronchial secretions of SARS-CoV-2 infected patients in a randomised, double-blinded and controlled study.

**Results:**

The dogs were able to discriminate between samples of infected (positive) and non-infected (negative) individuals with average diagnostic sensitivity of 82.63% (95% confidence interval [CI]: 82.02–83.24%) and specificity of 96.35% (95% CI: 96.31–96.39%)*.* During the presentation of 1012 randomised samples, the dogs achieved an overall average detection rate of 94% (±3.4%) with 157 correct indications of positive, 792 correct rejections of negative, 33 incorrect indications of negative or incorrect rejections of 30 positive sample presentations.

**Conclusions:**

These preliminary findings indicate that trained detection dogs can identify respiratory secretion samples from hospitalised and clinically diseased SARS-CoV-2 infected individuals by discriminating between samples from SARS-CoV-2 infected patients and negative controls. This data may form the basis for the reliable screening method of SARS-CoV-2 infected people.

## Background

The ongoing COVID-19 pandemic highlights the importance of fast and reliable testing for accurate identification of symptomatic and asymptomatic carriers to reduce spread of infection effectively [1]. Current testing regimens usually require nasopharyngeal swabs applied by a trained person and a reverse transcription polymerase chain reaction test (RT-PCR) for pathogen identification. Obtaining RT-PCR results is time consuming and can be cost-prohibitive, especially for developing countries, and is therefore currently often used in a targeted fashion, testing predominantly patients with COVID-19 specific symptoms [1]. There is therefore a need for an additional faster, reliable, non-invasive, and versatile screening tool, especially to identify asymptomatic and pre-symptomatic individuals.

Several studies have proven the canines’ extraordinary olfactory acuity to detect persons with infectious and non-infectious diseases like different types of cancer [2], malaria [3], bacterial, and viral infections [4–6], with usually high rates of sensitivity and specificity [7]. A pathogen-specific odour that can be detected by dogs may be composed of specific patterns of volatile organic compounds (VOCs). Compared to bacteria, viruses have no own metabolism, and therefore VOCs are released by infected body cells as a result of metabolic host processes [8]. Different technical approaches have used the detection of VOCs to discriminate infectious diseases successfully, but none is being used routinely in clinical practice [9]. As dogs can be trained quickly, the aim of the present study was to test the concept of using dogs reliably and in real-time to discriminate between samples of SARS-CoV-2 infected patients and non-infected controls. This method could be employed in public areas such as airports, sport events, borders or other mass gatherings as an alternative or addition to laboratory testing, thus helping to prevent further spreading of the virus or further outbreaks.

## Methods

### Sample acquisition

Saliva samples and tracheobronchial secretion samples were collected from hospitalised COVID-19 patients that showed clinical symptoms and were diagnosed as SARS-CoV-2 positive using nasopharyngeal swab samples. Negative control samples were obtained from SARS-CoV-2 RT-PCR negative people with no previous history of COVID-19, nor had the individuals any history of a recent cold or infection. None of the samples were screened for different human coronaviruses like beta coronavirus HCoV-OC43 or alpha coronavirus HCoV-229E. After the sample acquisition, the anonymised samples were transported to the *University of Veterinary Medicine Hannover*.

### Sample preparation

All collected samples were confirmed as positive or negative using the RT-PCR SARS-CoV-2-IP4 assay from *Institut Pasteur* (recommended by the *World Health Organization* [10, 11], including an internal control system and protocol as described [12, 13]. Samples from COVID-19 patients (irrespective of the final RT-PCR result) were further subjected to virus quantification (end point dilution assay) and virus isolation analysis using Vero E6 cells under biosafety level 3 conditions. The cell layers were assessed for cytopathic effects and final results were obtained 7 days after cell infection. Since dogs are susceptible to SARS-CoV-2 [14] all samples from COVID-19 patients were inactivated using beta propiolactone (BPL) in order to protect the dogs and their handlers from infection during training. Briefly, samples and reagents were kept at 4 °C, 20 μl/ml NaHCO_3_ (7.5%) was added, and samples were incubated for 10 min at 4 °C. After addition of 10 μl/ml of 10% BPL, samples were incubated at 4 °C for 70 to 72 h. Hydrolysis of BPL was conducted at 37 °C for 1 to 2 h. Samples that showed a cytopathic effect before BPL inactivation using virus isolation or end point dilution assay were tested again after BPL inactivation and were confirmed to be inactivated. Only BPL inactivated samples from COVID-19 patients were used for the dog training. Furthermore, detection dogs were provided both negative control samples with and without previous BPL treatment to exclude hydrolysed BPL as a potential distracting reagent.

For the dog training, a volume of 100 μl per sample was pipetted onto a cotton pad, which was placed into a 4 ml glass tube.

### Dog training and study design

The presentation of the samples to the dogs was conducted via a device called Detection Dog Training System (DDTS; Kynoscience UG, Germany), which can present samples in a randomised automated manner without trainer interference. For a short video sequence, see Additional file 1. DDTS was utilised for training and testing. The device is composed of seven scent holes. Behind each hole two tubes are leading to two metal containers. In the study, the first container enclosed the target sample and the second one carried the control sample. Only one container is presented in each sniffing hole at any given time as the pairs of containers are situated on movable slides inside the device. The metal containers were covered with grids, which allowed the odour to escape and reach the sniffing hole. Each tube extension was identical and L-shaped, which prevented dogs from physical contact with the samples and excluded any visual cues that may have enabled further detection capabilities. For each trial run, only one hole presented a SARS-CoV-2 positive sample at a time while the other six holes presented negative samples. After the indication of the hole with the positive sample, the dog was automatically rewarded by the device with food or ball. The indication time was changed during successful training from 1 s to 2 s. While the reward was eaten, the device’s software randomly and automatically assigned new positions to the slides for the following session with again only one hole presenting the positive odour sample.

The dog, its handler and a person observing the study were blinded during the double-blinded study. All personnel stood behind the dog during the test runs to avoid distraction. The device recorded automatically the number and time length of each nose dip into the scent holes and the location of the positive and negative samples. This was verified by manual time-stamped video analysis.

### Analysis of sensitivity and specificity

The diagnostic sensitivity (Se = true positive (‘TP’) /[TP + false negative (‘FN’)]), diagnostic specificity (Sp = true negative (‘TN’) /[TN + false positive (‘FP’)]), positive predictive values (PPV = TP/[TP + FP]) and negative predictive values (NPV = TN/[TN + FN]) were calculated according to Trevethan [15].

## Results

After a 2 weeks habituation process to the DDTS, the eight dogs needed 5 days of training in total until the detection rate was above chance. An additional spreadsheet provides background information of the dogs used in the study (see Additional file 2). The controlled double-blinded detection study was then conducted after 7 days of training and in total 10,388 sample presentations (Table 1).

**Table 1:** Number of presented samples per dog during training

Day	Sample status	Dog 1	Dog 2	Dog 3	Dog 4	Dog 5	Dog 6	Dog 7	Dog 8	Total
**1**	positive	15	20	15	15	15	15	15	10	120
	negative	90	120	90	90	90	90	90	60	720
**2**	positive	15	15	15	15	15	15	10	15	115
	negative	90	90	90	90	90	90	60	90	690
**3**	positive	35	35	35	35	30	35	38	35	278
	negative	210	210	210	210	180	210	228	210	1668
**4**	positive	20	15	20	20	20	40	60	20	215
	negative	120	90	120	120	120	240	360	120	1290
**5**	positive	40	40	30	30	35	30	53	60	318
	negative	240	240	180	180	210	180	318	360	1908
**6**	positive	20	7	20	14	10	10	30	15	126
	negative	120	42	120	84	60	60	180	90	756
**7**	positive	50	30	50	47	30	30	35	40	312
	negative	300	180	300	282	180	180	210	240	1872
**Total samples:**		1365	1134	1295	1232	1085	1225	1687	1365	10,388

On each training day, unknown and known positive samples and negative control samples were presented to the canines. The response to the new sample was used in order to evaluate if the generalisation process has been achieved. While the dogs had only achieved an average detection rate of 50% on the second day of training, the values increased to 70% on day five and even 81% on day seven indicating a successful generalisation process. After completion of the training process, the detection accuracy of the eight trained dogs was evaluated in a randomised, double-blinded, and controlled study (Table 2). Samples from seven infected and seven healthy individuals were used in this study. Two of the positive samples were tracheobronchial secretion, the other samples consisted of saliva.

**Table 2:** Diagnostic performance of the eight scent detection dogs

	Detect-ion by dog	SARS-CoV-2 infection status		Total number	Diagnostic specificity (Sp)	Diagnositic sensitivity (Se)	Standard Error (SE) Sp	Standard Error (SE) Se	Confidence Interval (95%CI) Sp	Confidence Interval (95%CI) Se	Negative predictive value (NPV)	Positive predictive value (PPV)	Standard Error (SE) NPV	Standard Error (SE) PPV	Confidence Interval (95%CI) NPV	Confidence Interval (95%CI) PPV
		negative	positive													
**Dog 1**	**No**	89	3	113	0.99	0.87	0.01	0.07	0.002	0.029	0.97	0.95	0.02	0.04	0.004	0.018
	**Yes**	1	20													
**Dog 2**	**No**	91	1	115	0.97	0.95	0.02	0.05	0.004	0.020	0.99	0.87	0.48	0.07	0.097	0.031
	**Yes**	3	20													
**Dog 3**	**No**	81	2	104	0.99	0.91	0.01	0.06	0.003	0.026	0.98	0.95	0.62	0.05	0.134	0.019
	**Yes**	1	20													
**Dog 4**	**No**	95	4	120	0.97	0.82	0.02	0.08	0.003	0.034	0.96	0.86	0.69	0.07	0.136	0.031
	**Yes**	3	18													
**Dog 5**	**No**	110	2	135	0.97	0.91	0.02	0.06	0.003	0.026	0.98	0.87	0.53	0.07	0.099	0.030
	**Yes**	3	20													
**Dog 6**	**No**	137	8	170	0.96	0.71	0.02	0.09	0.003	0.032	0.94	0.80	0.78	0.08	0.129	0.028
	**Yes**	5	20													
**Dog 7**	**No**	92	5	122	0.95	0.80	0.02	0.08	0.004	0.031	0.95	0.80	0.78	0.08	0.156	0.031
	**Yes**	5	20													
**Dog 8**	**No**	97	8	133	0.92	0.70	0.03	0.09	0.005	0.033	0.92	0.68	0.89	0.09	0.170	0.034
	**Yes**	9	19													
**Total**	**No**	792	33	1012	**0.96**	**0.83**	0.01	0.03	**0.0004**	**0.004**	0.96	0.84	1.18	0.03	0.081	0.004
	**Yes**	30	157													

**Fig. 1:**
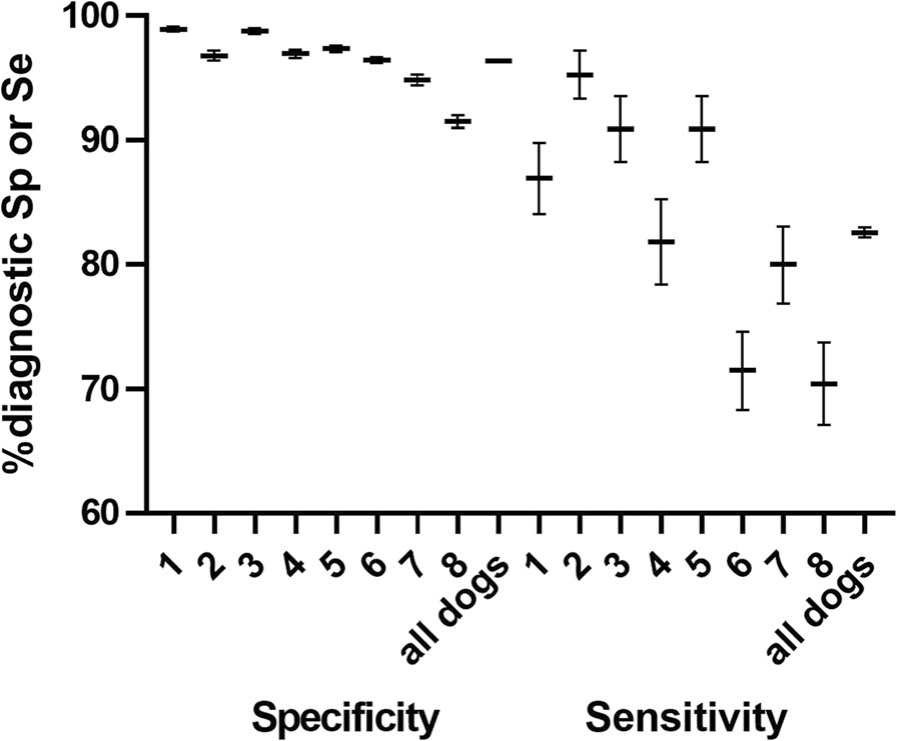
Diagnostic specificity and sensitivity by dog and for all dogs together. Whiskers show 95% confidence intervals

Within randomised and automated 1012 sample presentations, dogs achieved an overall average detection rate of 94% (±3.4%) with 157 correct indications of positive, 792 correct rejections of negative, 33 false positive and 30 false negative indications. The canines discriminated between infected and non-infected individuals with an overall diagnostic sensitivity of 82.63% (95% confidence interval [CI]: 82.02–83.24%) and specificity of 96.35% (95% CI: 96.31–96.39%). Sensitivity ranged from 67.9 to 95.2% and specificity from 92.4 to 98.9% (Fig. 1). There was no notable difference in detection ability between saliva and tracheal secretion (average hit rates 85.1 and 87.7%, respectively).

## Discussion

Timely and accurate detection of SARS-CoV-2 infected individuals is of uttermost importance for a society to control the pandemic. Our data indicate that detection dogs can be trained in just about a week to discriminate between samples of people infected and non-infected by SARS-CoV-2. The average detection rate was 94%. Analysis for accuracy and precision revealed a diagnostic sensitivity of 82.63% (95% CI: 82.02–83.24%) and a high diagnostic specificity of 96.35% (95% CI: 96.31–96.39%) for all dogs. All dogs had a high diagnostic specificity with a small range in variation, which could be important for population screening to avoid false positive results. However, there was quite a range in variation of sensitivity for the individual dog and inbetween dogs. This can in part be explained with the dogs’ variable training background (see Additional file 2), signalment, personality traits and short training period of 7 days. To avoid a bias concerning hospital specific smells, positive samples were obtained from two different hospitals to include a variation in a covariate factor and this appears to have not influenced the current results. Understanding better why there is this range in sensitivity and how to potentially improve it would be important prior to considering the use of detection dogs in the field. In comparison, the current gold standard diagnostic RT-PCR test of a nasopharyngeal swab can, in trained hands, have a false detection rate of 25% and a false positive rate of 2.3–6.9% [16]. A new, not yet published study indicated a clear, nearly 100% VOC specific pattern of SARS-COV-2 infected individuals compared to negative controls and individuals infected by the influenza virus using multicapillary column coupled ion mobility spectrometry of breath [17]. This provides further indications that unique VOC imprints exist and can be used for the development of diagnostic procedures.

The current study results are promising, although they should be regarded as preliminary and suitability for this detection method in the field can only be acquired after further research has been conducted. Our work provides the very first steps of the development of a new SARS-CoV-2 screening method. Our inclusion criteria for the samples collected were rather non-specific and not stratified by severity of symptoms, disease status or virus load. Future studies are needed to address this including a higher number of different samples to evaluate the analytical sensitivity (e.g. dilution of samples/detection level, different disease phenotypes and stages) and analytical specificity (differentiation to other lung diseases or pathogens such as cancer or infection with other seasonal respiratory virus infections, e.g. influenza, respiratory syncitial virus, adenovirus, other than SARS-CoV-2 coronaviruses, rhinovirus). In the current study negative control samples were acquired from healthy individuals without clinical signs of respiratory disease. The individuals were only tested for SARS-CoV-2 virus and therefore one cannot exclude that a former infection, especially with another human coronavirus like HCoV-OC43 resulted in false positive indications of the dogs and that cross detection occurred. On the other hand, samples included in the current study were from severely affected, hospitalised COVID-19 patients, but one of the main challenges in controlling the current pandemic is to identify pre-symptomatic COVID-19 patients and asymptomatic carriers, which may constitute most COVID-19 cases [18]. The sensitivity of detection by dogs may also vary across the course of the disease. Future research should therefore focus on the ability of dogs to identify the different COVID-19 disease phenotypes and phases of disease expression, such as asymptomatic, pre-symptomatic, mild and severe clinical cases as well as to test samples of the same individuals at different timepoints across the course of the disease.

One of the most important requirements regarding handling of infectious samples is infection prevention and control. Initially, it was assumed that dogs cannot get infected by SARS-CoV-2, but recent single cases have been reported showing that dogs can get infected by SARS-CoV-2 and could potentially play a role in viral spread [14, 19]. There is evidence of human-to-animal transmission with a subsequent infection of dogs. It is still unclear whether dogs can function as spreaders of the virus by infecting other animals or humans [14, 20]. Nevertheless, this needs to be considered when using dogs for detection of infected material or people. It is also unclear how an infection in the dog will alter its sense of smell. In the current study we chose to use an inactivation procedure which should not affect VOCs. However, this is not practical for testing in the field and we are currently developing new strategies for a secure presentation of non-inactivated samples. This would eliminate potential risks of virus transmission by detection dogs when used in a non-laboratory setting.

## Conclusions

Detection dogs were able to discriminate respiratory secretions of infected SARS-CoV-2 individuals from those of healthy controls with high rates of sensitivity and specificity. The current pilot study had major limitations which needs to be elucidated in future studies. SARS-CoV-2 detection dogs may then provide an effective and reliable infection detection technology in various settings like public facilities and function as an alternative or addition to regular RT-PCR screening. In countries with limited access to diagnostic tests, detection dogs could then have the potential to be used for mass detection of infected people. Further work is necessary to better understand the potential and limitation of using scent dogs for the detection of viral respiratory diseases.

## Supplementary information

**Additional file 1: Additional video.** Detection dog working with DDTS. The video (Additional file 1) shows the Labrador Retriever “Seven” during a detection session. The Detection Dog Training System (DDTS) can be seen at the bottom of the video. The scent hole with a sample of an SARS-CoV-2 infected individual is marked in green on the video (please note the green mark was not seen by the dog and was only used in the video as a visualisation tool for the viewer to demonstrate the dog’s search and detection behaviour). At each detection trial run only one hole is presenting the target scent with the other six holes presenting saliva samples from SARS-CoV-2 negative tested individuals. When the dog detects the target scent, the nose will be left within the hole for ≥2 s to indicate the detection. This will be recorded by the device. A beeping sound announces the food or ball reward, which is automatically ejected by the device, distracting the dog for a short time period. In the meantime, the device rearranges the sample presentation in an automatic and random fashion, presenting one other scent hole with a sample of a SARS-CoV-2 positive tested individual and six control scent holes with negative control samples. In the upper left corner of the video, one can see how the figures change depending on the detection behaviour of the dog (true positive [correct indication; *n* = 3], true negative [correct rejection; *n* = 8], false positive [incorrect indication; *n* = 0], and false negative [incorrect rejection; *n* = 1]).

**Additional file 2: Additional Table.** Characteristics of the dogs. The additional table (Additional file 2) shows the signalment and background of the eight dogs that participated in the study.

## Data Availability

The datasets used and/or analysed during the current study are available at Jendrny, Paula, Twele, Friederike, Schulz, Claudia, Meller, Sebastian, von Köckritz-Blickwede, Maren, Volk, Holger Andreas. (2020). SARS-CoV-2 detection dogs -a pilot study [Data set]. Zenodo. 10.5281/zenodo.3950074

## Abbreviations

RT-PCR: reverse transcription polymerase chain reaction test
BPL: beta propiolactone
VOCs: volatile organic compounds
DDTS: Detection Dog Training System
CI: confidence interval
TP: true positive
FN: false negative
Sp: specificity
TN: true negative
FP: false positive
PPV: positive predictive values
NPV: negative predictive values
